# Utilizing the MEST score for prognostic staging in IgA nephropathy

**DOI:** 10.1186/s12882-021-02653-y

**Published:** 2022-01-11

**Authors:** Yngvar Lunde Haaskjold, Rune Bjørneklett, Leif Bostad, Lars Sigurd Bostad, Njål Gjærde Lura, Thomas Knoop

**Affiliations:** 1 Renal Research Group, Department of Clinical Medicine, University of Bergen, Bergen, Norway; 2 Department of Medicine, Haukeland University Hospital, 5021 Bergen, Norway; 3 Emergency Care Clinic, Haukeland University Hospital, Bergen, Norway; 4 Department of Pathology, Haukeland University Hospital, Bergen, Norway; 5 Department of Radiology, Haukeland University Hospital, Bergen, Norway

**Keywords:** IgA nephropathy, Prognosis, Prediction model, Kidney biopsy, MEST score

## Abstract

**Background:**

The Oxford classification/MEST score is an established histopathologic scoring system for patients with IgA nephropathy (IgAN). The objective of this study was to derive a prognostic model for IgAN based on the MEST score and histopathologic features.

**Methods:**

A total of 306 patients with biopsy-proven primary IgAN were included. Histopathologic samples were retrieved from the Norwegian Kidney Biopsy Registry and reclassified according to the Oxford classification. The study endpoint was end-stage renal disease (ESRD). Patients were subclassified into three risk models based on histologic features (Model A), a composite score calculated from the adjusted hazard ratio values (Model B), and on quartiles (Model C).

**Results:**

The mean follow-up time was 16.5 years (range 0.2–28.1). In total, 61 (20%) patients reached ESRD during the study period. Univariate analysis of M, E, S, T and C lesions demonstrated that all types were associated with an increased risk of ESRD; however, a multivariate analysis revealed that only S, T and C lesions were associated with poor outcomes. Statistical analysis of 15-year data demonstrated that Models A and B were as predictive as the MEST score, with an area-under-the-curve at 0.85. The Harrel c index values were 0.81 and 0.80 for the MEST score and Models A and B, respectively. In the present cohort, adding C lesions to the MEST score did not improve the models prognostic value.

**Conclusions:**

Patients can be divided into risk classes based on their MEST scores. Histopathologic data provide valuable prognostic information at the time of diagnosis. Model B was the most suitable for clinical practice because it was the most user-friendly.

**Supplementary Information:**

The online version contains supplementary material available at 10.1186/s12882-021-02653-y.

## Introduction

As with most countries, immunoglobulin A nephropathy (IgAN) is the most frequently diagnosed primary glomerulonephritis in Norway [1]. The clinical course of IgAN is variable, with some patients progressing to end-stage renal disease (ESRD), whereas others maintain stable renal function over the long term [2]. IgAN is the leading cause of ESRD in young adults and is associated with significant personal and socioeconomic losses. There is currently no effective medical treatment besides supportive care. However, a number of recent therapeutic clinical trials are examining novel treatment options because of novel insights into the complex pathogenesis of IgAN [3]. These new treatments emphasize the importance of accurate prediction tools that can stratify patients according to specific types of therapy [4–6].

A kidney biopsy is mandatory for the diagnosis of IgAN and is also an important source of prognostic information [2]. In 2009, an international consensus group developed the Oxford classification, which is a histopathological tool that aims to predict prognosis in patients with IgAN independent of clinical data [7, 8]. The Oxford classification describes four histopathological lesions associated with adverse outcomes. It is well established and has been validated by numerous studies [9–18]. The original Oxford classification examined mesangial hypercellularity (M), endocapillary hypercellularity (E), segmental glomerulosclerosis (S), and tubular atrophy/interstitial fibrosis (T) [7, 8] and reported this as the MEST score. In 2016, crescents (C) was added to the original classification [19]. The Oxford classification is now a part of the routine evaluation practice in Norway, but many nephrologists without special interests in IgAN find it difficult to interpret the results and apply the prognostic model in their clinical practice. Potentially the MEST score can report 32 different combinations: simplifying the way results are reported could increase the clinical usefulness of this prognostic model.

In the present study, we sought to convert the MEST score into a risk model with 4–6 risk classes instead of 32, enabling pathologist to provide clinicians with basic information of prognosis in the pathology report. To establish the risk classes, we reclassified biopsies according to the Oxford classification in 306 patients with IgAN, retrieved from *The Norwegian Kidney Biopsy Register* (NKBR). The study cohort had moderate risk for ESRD, with estimated glomerular filtration rate (eGFR), CKD-EPI formula, above 30 mL/min/1.73 at time of biopsy and up to 28 years of follow-up.

## Materials and methods

### Ethics

The study was approved by the Western Norwegian Regional Committee for Medical and Health Research Ethics (Reference no. 2018/2130). All the study participants provided informed consent.

### Data collection

The Norwegian Kidney Biopsy Registry was founded in Bergen in 1988. It contains the clinical, biochemical, immunologic, and morphologic data from patients subject to kidney biopsy in Norway since April 1988. The Norwegian Renal Registry is located in Rikshospitalet in Oslo. This registry contains the data of all patients with ESRD who received dialysis or underwent a kidney transplant in Norway since 1980.

### Study population

Patients registered in NKBR with biopsy-proven IgAN prior to 2010, initial estimated eGFR above 30 mL/min/1.73 m^2^, and histopathological specimens available for re-analyses were included. Patients with secondary forms of IgAN, including IgA Vasculitis (IgAV) were excluded from the study. Cases with ESRD during follow-up was identified by record linkage with the Norwegian Renal Registry. The resulting data set was complemented by retrieving supplementary data from patient records.

### Observation period

All patients were observed from the time of the diagnostic kidney biopsy until ESRD, death, or the termination of the study on April 2020 was reached.

### Study endpoint

ESRD was defined as the commencement of maintenance dialysis or receiving a kidney transplant.

### Histopathological studies

The original biopsy slides, examined under hematoxylin-eosin, periodic acid-Schiff, periodic methenamine silver, Masson’s trichrome, and immunohistochemistry stains, were retrieved from the Norwegian Kidney Biopsy Registry. An experienced renal pathologist, who was blinded for clinical information, did a complete microscopic review. The pathologic variables were scored according to the Oxford classification MEST-C criteria as follows: mesangial hypercellularity (M0/M1), endocapillary hypercellularity (E0/E1), segmental glomerulo- sclerosis (S0/S1), tubular atrophy/interstitial fibrosis (T0/T1/T2), and crescents (C) [7, 8, 19]. A small minority of the slides lost their color and were re-stained.

### Statistics

The baseline characteristics of patients with and without progression to ESRD were compared; the Student’s T-test and chi-square test were used to evaluate continuous and categorical variables, respectively. Cumulative survival without ESRD, stratified according to the MEST score and risk groups/models, was calculated using the Kaplan-Meier curve. The log-rank test was used to assess for statistical significance. A Cox regression analysis was performed to calculate for unadjusted and adjusted hazard ratios (HRs) between ESRD and each criterion of the MEST score. The adjusted analysis accounted for other factors in the MEST score, as well as for the use of immunosuppressants. Using the adjusted HRs, we calculated a composite risk score for the MEST score. The weight of each of the criteria in the MEST score was derived from the adjusted HRs estimated by a Cox proportional hazard model. The composite score was calculated using the following formulae: HR M0, HR M1 × HR E0, HR E1 × HR S0, HR S1 × HR T0, or HR T1 (HR = adjusted HR). Cumulative discriminative performance for predicting ESRD at 15 years of follow up, was evaluated with a time-dependent Receiver Operating Characteristics (ROC) analysis. A discriminatory global concordance summary was provided by C-statistics. Model calibration for MEST and Model B was evaluated by comparing observed and predicted events in calibration plots using the resampling calibration function in the rms-R package. Model fit for both models was evaluated with the Akaike information criterion (AIC). The discriminatory performance of MEST and Model B was internally validated using the optimism boot strap method. 1000 bootstrap samples were constructed, the models were refitted for each boot strap sample, and model performance evaluated in both bootstrap sample and original sample. The optimism estimate was calculated as the mean difference of these performance estimates. Evaluation of the suggested models were done according to the TRIPOD recommendations [20]. Statistical analysis was performed in R Statistics 1.2.1335 (Vienna, Austria) and, IBM SPSS Statistics for Windows, Version 25.0 (IBM Corporation, Armonk, New York).

### Development of risk models

We utilized three different approaches to convert the MEST scores into risk models.

Model A was a histological chronicity model based on the hypothesis that progression in IgAN follows a sequential pattern. Three major risk groups were created based on the S and T status, particularly S0/T0, S1/T0, and S1/T1. We further divided these risk groups according to the M and E status; M0E0 versus M1/E0, M0/E1, or M1/E1. This model could not classify the three patients in the study cohort with S0/T1 scores. We applied a mathematical model to Model B using the composite risk score described in the methods section. Risk group 1, score 1.0; risk group 2, score 1.1–4; risk group 3, score 5.0–14.9; and risk group 4, score ≥ 15.

Model C analyzed the composite risk score as four quartiles of patients. First, we sorted the cohort according to rising composite risk score. Second, we divided the cohort into 4 similar size groups, each containing 76 or 77 patients. Third, while prioritizing best possible similar sized quartiles we moved patients with similar composite risk scores into the same risk groups. However, as a result the quartiles were not exactly of similar size.

## Results

### Clinical and histopathological characteristics

In total, 306 patients were included in the study. Two hundred thirty-four (76.5%) patients were male. The mean age at the time of biopsy was 37.4 years. The mean and median patient follow-up time was 16.5 years (range 0.2–28.1) and 17.1 years (range 0.16–28.1) respectively. The median follow-up time for patients reaching ESRD was 7.93 years and 19.0 years for patient without progression to ESRD. At the time of biopsy, the mean eGFR was 78.4 mL/min/1.73 m^2^, whereas the mean proteinuria was 1.7 g/day. The majority of the patients (70.9%) were on renin-angiotensin aldosterone-system (RAAS) blockade therapy. Immunosuppressants were used by 20 (6.5%) of the patients.

M1 was observed in 33.7% of the biopsies, E1 in 26.5%, and S1 in 54.9%. T-lesions were observed in 11.1% of the biopsies. There were only two cases of T2 lesions, and T1-T2 were merged to simplify statistical analysis. Further clinical and histopathological data at baseline are listed in Table 1.

**Table 1:** Baseline characteristics all patients and stratified for ESRD during follow-up

Characteristics	All = 306	Not ESRD = 245	ESRD = 61	***P***-value
Duration of follow-up (years), mean (SD) ^a^	16.6 (7)	18.5 (5)	8.8 (6)	
Age at biopsy (years), mean (SD)	37.4 (14)	37.2 (14)	38.0 (13)	0.67
Male gender (%)	234 (76.5%)	182 (74.3%)	52 (85.2%)	0.07
M = 1 (%)	103 (33.7%)	72 (29.4%)	31 (50.8%)	0.002
E = 1 (%)	81 (26.5%)	55 (22.4%)	26 (42.6%)	0.001
S = 1 (%)	168 (54.9%)	116 (47.3%)	52 (85.2%)	<0.001
T = 1-2 (%)	34 (11.1%)	10 (4.1%)	24 (39.3%)	<0.001
C = 1 (%)	61 (19.9%)	39 (15.9%)	22 (36.0%)	<0.001
C = 2 (%)	9 (2.9 %)	5 (2.0%)	4 (6.6%)	<0.001
eGFR (mL/min/1.73 m^2^), mean (SD)	78.4 (27)	83.9 (26)	56.3 (23)	<0.001
Proteinuria (g/day), mean (SD)	1.7 (2.3)	1.3 (1.9)	3.4 (2.8)	<0.001
Systolic BP (mmHg), mean (SD)	135 (17)	134 (17)	139 (18)	0.03
Diastolic BP (mmHg), mean (SD)	83 (11)	82 (11)	88 (10)	0.001
RAAS (%)^b^	217 (70.9%)	159 (64.9%)	58 (95.1%)	<0.001
Immunosuppressives (%)^c^	20 (6.5%)	10 (4.1%)	10 (16.4%)	<0.001

^a^Follow-up stop at time of ESRD

^b^ACE-inhibitors or angiotensin II blockers

^c^Corticosteroids or other immunosuppressive drugs

**Fig. 1:**
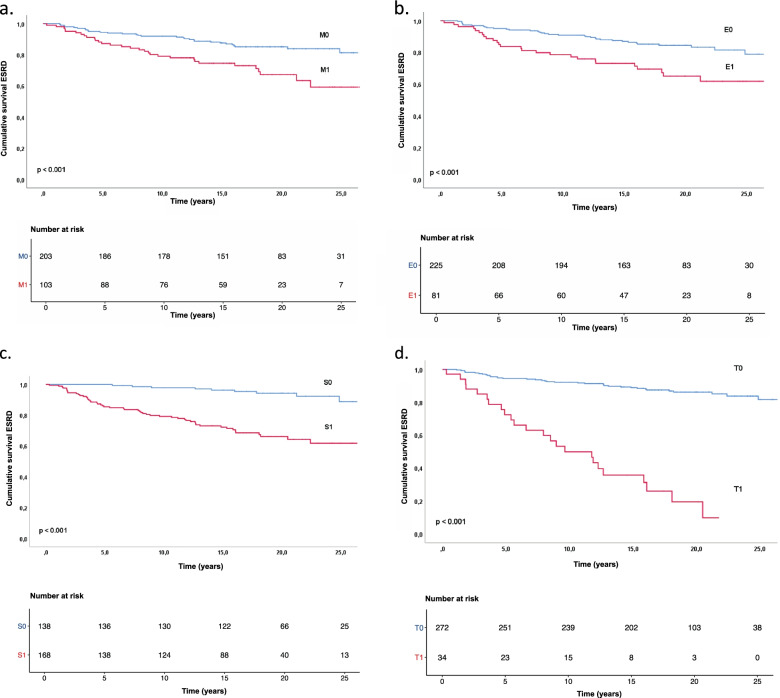
**a**-**d** Kaplan-Meier plots showing cumulative risk of end stage renal disease (ESRD) in the different factors in the MEST score: M (**a**), E (**b**), S (**c**) and T (**d**)

**Fig. 2:**
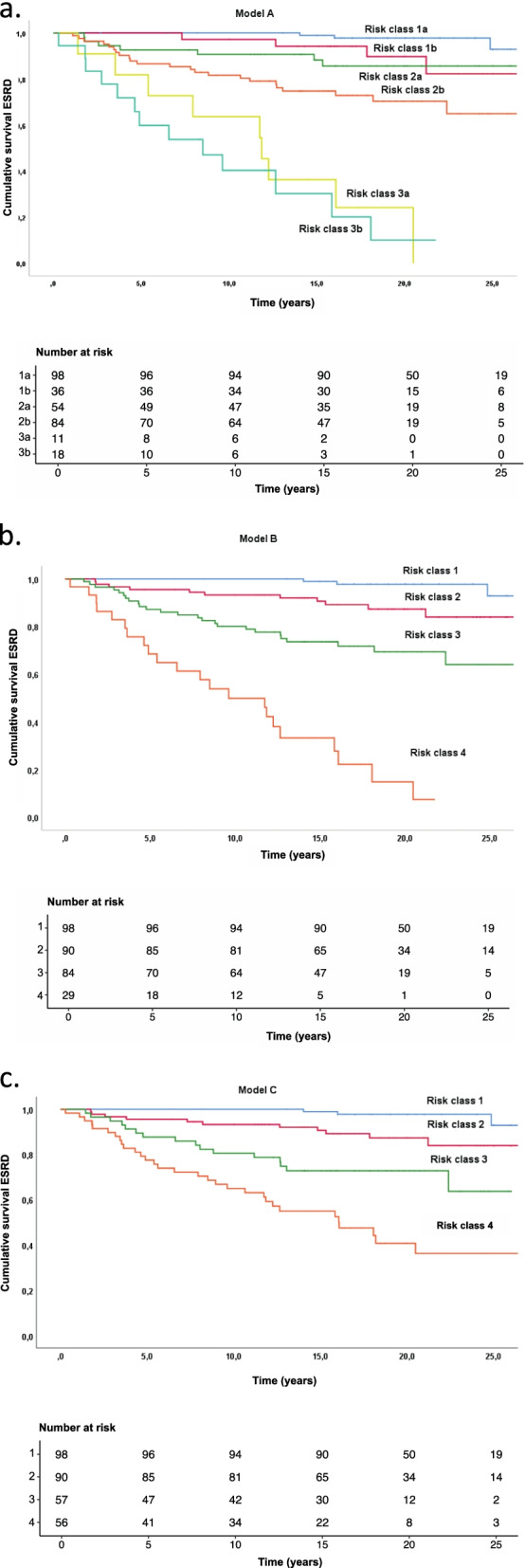
**a**-**c** Kaplan-Meier plots showing cumulative risk of end stage renal disease (ESRD) in the different risk models: Model A (**a**), model B (**b**) and model C (**c**)

In total, 61 (20%) patients reached ESRD during the study period. Table 1 shows that the patients who reached ESRD had significantly lower eGFR, more proteinuria, and higher systolic and diastolic blood pressure at the time of biopsy. The cumulative risk for ESRD at 5, 10, 15, 20 and 25 years were 8.0, 12.4, 16.8, 20.7, and 25.6 respectively. We analyzed the same data stratified for factors in the MEST score and risk models. These data are shown in Table 2 and Figs. 1 and 2.

**Table 2:** Cumulative risk of ESRD stratified for MEST C classification

Characteristics	N	ESRD	Cumulative risk of ESRD				
			5 years	10 years	15 years	20 years	25 years
All	306	61	8.0	12.4	16.8	20.7	25.6
M0	203	30	5.5	8.1	12.4	14.9	18.7
M1	103	31	12.8	20.9	25.5	32.9	40.8
E0	225	35	5.0	9.1	13.1	15.6	21.1
E1	81	26	16.3	21.5	27.0	35.0	38.3
S0	138	9	0.0	2.2	3.8	5.7	11.3
S1	168	52	14.6	20.9	27.8	33.9	38.3
T0	272	37	5.6	7.9	11.1	13.8	18.4
T1-T2	34	24	27.7	50.2	64.4	80.5	90.3
C0	236	35	3.9	7.0	11.7	15.3	19.9
C1	61	22	18.0	27.9	31.3	37.5	46.4
C2	9	4	50.0	50.0	50.0	50.0	50.0

### MEST scores and outcomes

Univariate analysis demonstrated that M, E, S, and T lesions were all associated with an increased risk for ESRD, whereas a multivariate analysis demonstrated that only S and T lesions were significantly associated with poor outcomes, with HRs of 3.76 and 6.43 respectively. Further data are listed in Supplementary Table 1.

### MEST and models A–C

**Fig. 3:**
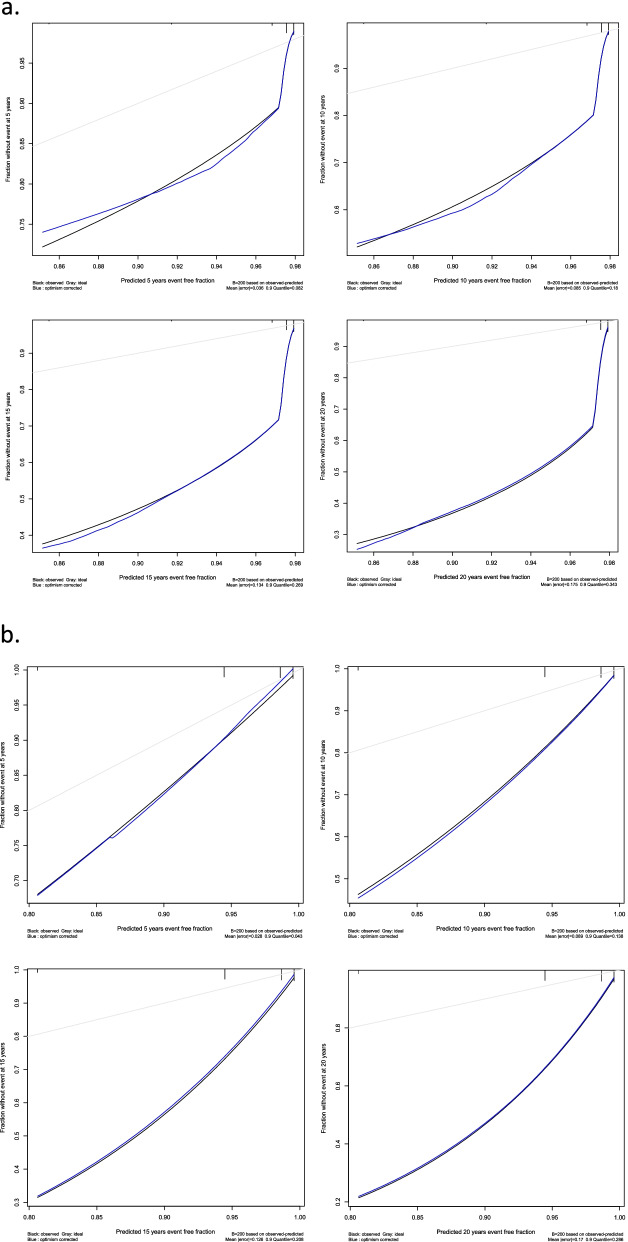
**a**-**b** Calibration curves with mean error and 0.9 quantile for MEST and Model B at 5, 10, 15 and 20 years

Despite being developed differently, the three models provided relatively similar results. Model A consisted of six risk classes, whereas Models B and C had four risk classes. However, Risk Classes 1b/2a and 3a/3b in Model A were identical to Risk Class 2 and 4 in Model B respectively. Further, risk class 1a and 2b in model A were equal to risk class 1 and 3 in model B. The sole difference between models B and C was that patient with an M1E1S1T0 pattern were categorized in risk class 3 in model B, but in risk class 4 in Model C. The combination of MEST scores subclassified according to risk class is shown in Table 3. The number of patients for each of the possible MEST score combinations in model B are listed in Table 4. The cumulative risk of ESRD for each model is shown in Fig. 2. Table 5 describes the cumulative risk for each of the risk classes in Model B. The ROC analysis compared MEST scores at 15 years with Models A–C and showed similar predictive values, with area-under-the-curve (AUC) scores of 0.85 for Models A and B, and 0.84 for Model C. The Harrel c index was 0.81 for the MEST score, 0.80 for Models A and B, and 0.79 for Model C, as listed in Table 6. The internal validation of MEST and Model B provided no significant optimism corrections for the models discriminatory performance. Calibration analysis for MEST and Model B showed that both models are well calibrated in the first 10 years, however the models tend to assign lower probabilities of event to patients with higher risk at later stages (Fig. 3a-b). AIC for was 580.93 and 553.40 for MEST and Model B respectively.

**Table 3:**
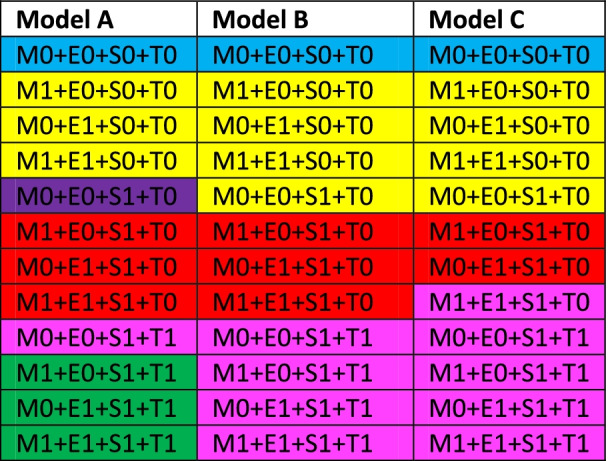
Combination of MEST score in the different models^a,b^

^a^ Combinations: M0+E0+SO+T1, M1+E0+S0+T1 and M1+E1+S0+T1 were left out from the model due to few (<2) cases in the study cohort

^b^ Risk classes representing model A are shown in brackets

**Table 4:** Number of patients for each of the possible MEST score combinations in model B

MEST score	Risk class	No. of Patients
0-0-0-0	1	98
1-0-0-0	2	16
0-1-0-0	2	11
1-1-0-0	2	9
0-0-1-0	2	54
1-0-1-0	3	33
0-1-1-0	3	24
0-0-0-1	3	2
1-1-1-0	3	28
1-1-0-1	4	1
0-0-1-1	4	12
1-0-1-1	4	9
0-1-1-1	4	2
1-1-1-1	4	7

**Table 5:** Cumulative risk of ESRD in different Risk classes in model B

Risk class	N	ESRD	Cumulative risk of ESRD				
			5 years	10 years	15 years	20 years	25 years
1	98	3	0.0	0.0	1.1	2.2	7.1
2	90	11	4.4	6.7	9.4	12.7	16.0
3	88	25	12.8	20.0	26.4	30.7	36.0
4	30	22	31.6	50.0	66.7	85.2	92.6

**Table 6:** Prognostic value of different models shown as AUC^a^ and Concordance index^b^

Model	AUC 5 yr.	AUC 10 yr.	AUC 15 yr.	AUC 20 yr.	C-index
MEST	0.84	0.85	0.85	0.85	0.81
Model A	0.83	0.85	0.85	0.85	0.80
Model B	0.82	0.84	0.85	0.85	0.80
Model C	0.80	0.82	0.83	0.84	0.79

^a^Area under the receiver operating characteristic curve

^b^Harrell’s concordance index (C-index)

## Discussion

In the present study, we confirmed that the MEST score is a precise and easy applicable prognostic tool for IgAN. Moreover, we demonstrate that it is feasible to simplify the score into different risk classes without significant loss of prognostic value. To the best of our knowledge, this is the first study that attempted to make the MEST score more simple.

In the recent two decades, several predictive scoring systems for patients with IgAN have been proposed. The majority of these scoring systems investigated the renal outcome (ESRD) as an endpoint by utilizing a predefined set of clinical and histopathological variables from the time of the diagnostic renal biopsy [21–24]. However, the prognostic value of these models has been difficult to compare, because of differences in follow-up duration, selected endpoints, selected variables as risk factors, and overall methodological approach. As such, most of these prognostic models have not been adopted into clinical practice due to the lack of generalizability and questionable clinical applicability. Moreover, only a few of these models have undergone external validation studies, which illustrates that IgAN is a heterogeneous disease; developing a satisfactory and universal prognostication model is challenging from clinical and research perspectives. An ideal predictive model would be useful in the clinical setting for individual counseling but also contribute to the scientific decision-making process, particularly in terms of assessing healthcare costs and selecting the inclusion criteria for randomized controlled trials.

In the present study, we applied three different approaches to develop risk models based on MEST scores. We understand that crescents (C) have been added to the Oxford classification [19, 25]. Haas et al. found that adding C lesions could improve the prognostic value in patients not treated with immunosuppressives [25]. Even though uni- and multivariate analysis (Supplementary Table 1) showed that C lesions had prognostic value, our data demonstrated that MEST and MEST-C scores have almost identical prognostic values. Accounting for C0–2 lesions in the risk classes would make the models more complicated because the number of possible combinations would increase threefold. As such, we opted to exclude C lesions from our models. Barbour et al. excluded C lesions from the International IgAN Prediction Tool arguing that their model included additional variables that Haas et al. did not adjust for in their analyses, and that use of immunosuppression could influence the primary endpoint. Further on, Barbour suggests that adding race to his model would diminish the predictive value of crescents [26]. We have the same strict inclusions criteria as the original Oxford study from 2009 [8], but the role of crescents could be even more prominent if patients with more severe disease were included [25].

Model A is based on the hypothesis that progression in IgAN follows a sequential pattern; nephropathy begins with the glomerular deposition of underglycosylated IgA molecules, which prompts mesangial hypercellularity (M) and/or endocapillary hypercellularity (E). Segmental sclerosis (S), global sclerosis, or tubular atrophy (T) develop in the long term.

In contrast, Models B and C are based on statistical logic and were developed from the results of the Cox regression analysis. Despite these different approaches, the resulting models demonstrated relatively similar results. In the respective cohort, Model B seemed the most attractive. Model A had six risk classes; however, the prognosis of Risk Classes 1b/2a and 3a/3b were similar. When these classes are merged model A and B are identical. The only difference between models B and C was that the MEST score of M1E1S1T0 was classified under Risk Class 3 in Model B but under Risk Class 4 in Model C. Since the M1E1S1T0 score was associated with significantly better outcomes than the S1T1 scores (data shown in supplementary), model B performed slightly better than model C.

Both the MEST score and Model B presented similar AUC and concordance indices, indicating that each model discriminated between low- and high-risk patients well. Model B is easy to apply and could provide clinicians with useful prognostic information. Model B is also based on HRs in a cohort with long follow-up times. As such, the HR may be different in a cohort with shorter follow-up times. We speculate that the impact of the factors in the MEST score could be dependent on the observation time, and, therefore, that M lesions could be a precursor to S and/or T lesions. The present study also only examined patients with biopsy-confirmed IgAN and eGFR values > 30 mL/min/1.73 m^2^. This can explain why C lesions lacked prognostic value and why T2 lesions were infrequent in our cohort. This decision was based on previous observations that patients with IgAN with eGFR values < 30 mL/min/1.73 m^2^ at presentation have a poor prognosis, with the exception of cases with acute reversible renal failure [27, 28]. Therefore, we propose that the benefits of a histopathological prognostic model for this group are limited. Multivariate analysis also demonstrated that S and T lesions were independent predictors for progressive decline in renal function among patients with IgAN. However, our model also highlighted that M and E lesions could distinguish between Risk Classes 1 and 2, and were therefore essential in determining the prognostic value of the composite MEST score. This contrasts with previous studies, which showed that E lesions have limited prognostic value and were more of indicators for the response to immunosuppressive treatment [17, 19, 29]. Only a small number of patients (6.5%) received immunosuppressants in our cohort, which could be a result of inclusion criteria limited to patients with mild to moderate disease. When we adjusted for immunosuppressive treatment, the prognostic value of E lesions demonstrated a non-significant decrease in the HR from 1.4 to 1.2, whereas the prognostic value of M, S, and T lesions did not change significantly. We, therefore, chose not to include immunosuppressive treatment in our model.

Efforts have been made to approach an ideal prognostic model that is both accurate and clinically relevant. Barbour et al. demonstrated that combining the MEST score with clinical data could improve risk prediction in IgAN [30]. Barbour et al., with Schena et al.*,* have also proposed prognostic models that combine histopathological and clinical data [4, 5]. Schena et al. further proposed a clinical decision support system based on an artificial neural network that can calculate the total quantitative risk for ESRD in IgAN up to 10 years [4]. Barbour et al. derived a prediction model for disease progression in IgAN up to 7 years that can be applied at the time of kidney biopsy and across multiple ethnic groups [5]. It is suggested that the 2020 Kidney Disease: Improving Global Outcomes guidelines encourage clinicians to use Barbour’s model for risk stratification, but not yet as a tool for selecting which patients will benefit from immunosuppressive treatment [31]. Interestingly, our ROC analysis demonstrated that a model that is based purely on histopathological data has almost the same predictive performance as the models that combine clinical and histopathological information. Recently, Miyabe et al., has demonstrated that the MEST C score could be utilized to predict prognosis in IgAN using a grading system were patients were classified into three groups, but the model has yet to be externally validated [32].

As previously noted, a good prognostic model combines precision and ease of use. The risk class model is user-friendly and enables pathologists to provide clinicians with reliable prognostic information at the time of diagnosis based on histology alone. Prognostic models that incorporate multiple clinical and histopathological values may provide higher levels of precision, but could also be more vulnerable to fluctuating and/or modifiable factors, such as proteinuria and blood pressure. For example, the use of RAAS-blocking agents could reduce blood pressure, proteinuria, and eGFR. Certain predictors can indeed add predictive value to a model, however more is not always better, and the price of adding more variables to a prediction model are added risk of overfitting and make it less user-friendly [33]. Our model converts the MEST score into simpler, more understandable data without any loss in prognostic precision. Moreover, the internal validation of the models shows that there was no tendency toward overfitting, bearing great promise for similar performance levels in the case of an external validation. Current models provide accurate estimates of short-term outcomes, but they are not validated for long-term use. Approximately 50% of the patients in our cohort reached ESRD later than 8 years after diagnosis. Future studies should address this by attempting to create prediction models with higher accuracy in long term prediction, since many patients with IgAN are afflicted at a young age and will potentially live with their disease for most of their life span.

There is currently no effective treatment for IgAN besides optimized supportive care, but immunosuppressive drugs may be considered in patients who demonstrate rapid progression to ESRD [34]. Reduction in proteinuria was recently approved by regulators as a surrogate endpoint for IgAN [35]; several clinical trials on proteinuria have since been initiated, bringing hope that an effective treatment may be within reach [3]. Future treatment emphasizes the importance of accurate prediction tools that can identify patients that might benefit from a specific therapy [6]. Several other morphological features, such as glomerular complement deposition [36] and macrophage count [37], have been identified as potential histopathological prognostic markers in IgAN. However, the lack of other robust biomarkers emphasizes the importance of kidney biopsies for prognostic information. It has been suggested that repeated kidney biopsies may provide clinicians with more accurate and up-to-date prognostic predictions [38]. Recent improvements in prognostic models, combined with developments in treatment alternatives, stress the importance of performing a kidney biopsy in patients with suspected IgAN [39].

The main limitations of this study are its relatively small sample size and retrospective study design. The lack of certain combinations of the MEST scores in the study cohort emphasizes the importance to explore the model in larger cohorts. However, this study also followed the participants over a long period of time, which enabled the use of a robust endpoint of ESRD compared to more uncertain surrogate endpoints, such as a > 50% decline in eGFR, the doubling of serum creatinine, or the rate of eGFR decrease, which have been used in other studies [19]. The data used in our study was also extracted from reliable sources. Additional studies can be performed on the same data to follow the course of treatment.

In conclusion, this study found that patients can be divided into risk classes based on their MEST scores. This creates a model that allows pathologists to provide clinicians with valuable prognostic information. We also demonstrated that the MEST score is a robust histopathological tool in predicting outcomes in patients with IgAN. The findings in this study should be verified with data from other cohorts [40].

## Supplementary Information

**Additional file 1.**

## Data Availability

The data underlying this article cannot be shared publicly due to Norwegian regulations and the privacy of individuals that participated in the study. The data could be shared on reasonable request to the corresponding author if accepted by Regional Committee for Medical and Health Research Ethics and local Data Protection Official.

## Abbreviations

AIC: Akaike information criterion
eGFR: Eestimated Glomerular Filtration Rate
ESRD: End Stage Renal Disease
HR: Hazard Ratio
IgAN: IgA Nephropathy
IgAV: IgA Vasculitis
NKBR: The Norwegian Kidney Biopsy Register
ROC: Receiver Operating Characteristics
