# Supplementary material for: Utilizing the MEST score for prognostic staging in IgA nephropathy
Source: BMC Nephrol. 2022 Jan 11;23:26. doi: 10.1186/s12882-021-02653-y (PMC8753851; doi:10.1186/s12882-021-02653-y)
Supplement: Supplementary file 1 — Additional file 1. [file 12882_2021_2653_MOESM1_ESM.docx]

**SUPPLEMENTARY CONTENT**

**UTILIZING THE MEST SCORE FOR PROGNOSTIC STAGING IN IGA NEPHROPATHY**

Yngvar Lunde Haaskjold, MD,^1, 2^ Rune Bjørneklett, MD, PhD,^1, 3^ Leif Bostad, MD PhD,^1, 4,^ Lars Sigurd Bostad, MD,^1, 3^, Njål Gjærde Lura, MD,^5^ , and Thomas Knoop, MD, PhD,^1, 2^

^1^ Renal Research Group, Department of Clinical Medicine, University of Bergen, Norway

^2^ Department of Medicine, Haukeland University Hospital, Bergen, Norway

^3^ Emergency Care Clinic, Haukeland University Hospital, Bergen, Norway

^4^ Department of Pathology, Haukeland University Hospital, Bergen, Norway

^5^ Department of Radiology, Haukeland University Hospital, Bergen, Norway

**Corresponding author:**

Yngvar Lunde Haaskjold, MD

Department of Medicine, Haukeland University Hospital, 5021 Bergen, Norway

Telephone +47 55974402

Email: yngvar.lunde.haaskjold@helse-bergen.no

**List**

**Supplementary table 1.** Multiple Cox regression analyses for ESRD in patients with IgA nephropathy

**Supplementary figure 1.** ROC analysis at 15 years comparing MEST and MEST-C score.

**Supplementary figure 2.** Kaplan-Meier curve comparing outcome in M1E1S1T0 and S1T1 score.

**Supplementary table 1.** Multiple Cox regression analyses for ESRD in patients with IgA nephropathy

| **Characteristics** | **N** | **ESRD** | **Unadjusted HR** | **Adjusted HR^a^** | **Adjusted HR^b^** |
| --- | --- | --- | --- | --- | --- |
|  |  |  |  |  |  |
| M0 | 203 | 30 | 1.0 | 1.0 | 1.0 |
| M1 | 103 | 31 | 2.38 (1.44-3.95) p= 0.001 | 1.39 (0.81-2.38) p=0.23 | 1.30 (0.76-2.21) p=0.34 |
| E0 | 225 | 35 | 1.0 | 1.0 | 1.0 |
| E1 | 81 | 26 | 2.35 (1.41-3.90) p=0.001 | 1.53 (0.89-2.62) p=0.12 | 1.25 (0.72-2.16) p=0.43 |
| S0 | 138 | 9 | 1.0 | 1.0 | 1.0 |
| S1 | 168 | 52 | 6.30 (3.10-12.81) p<0.01 | 3.76 (1.78-7.93) p=0.001 | 3.60 (1.71-7.56) p=0.001 |
| T0 | 272 | 37 | 1.0 | 1.0 | 1.0 |
| T1-T2 | 34 | 24 | 9.72 (5.70-16.58) p<0.001 | 6.43 (3.73-11.07) p<0.001 | 6.33 (3.66-10.94) p<0.001 |
| C0 | 236 | 35 | 1.0 |  | 1.0 |
| C1 | 61 | 22 | 3.20 (1.86-5.49) p<0.001 |  | 1.70 (0.96-3.00) p=0.07 |
| C2 | 9 | 4 | 7.08 (2.49-20.19) p<0.001 |  | 17.24 (4.94-60.11) p<0.001 |

N, number of patients; HR, hazard ratio; M, mesangial hypercellularity; E, endocapillary hypercellularity;

S, segmental sclerosis; T, interstitial fibrosis/tubular atrophy; C, crescents

^a^ Adjusted for other factors in MEST

^b^ Adjusted for other factors in MEST-C

**Supplementary figure 1.** ROC analysis at 15 years comparing MEST and MEST-C score.

MEST (red): AUC (%): 85.05, se: 2.84, CI: 79.49 - 90.61 (2.5% - 97.5%).

MEST-C (blue): AUC (%) 86.30, se 2.80, CI: 80.82 - 91.79 (2.5% - 97.5%).

P-value: 0.3362686.

**Supplementary figure 2.** Kaplan-Meier curve comparing outcome in M1E1S1T0 and S1T1 score.
